# Systemic Inflammatory Indices and Hearing Loss in U.S. Adults Aged 40–85 Years: A Serial Cross‐Sectional Study 1999–2020

**DOI:** 10.1002/iid3.70576

**Published:** 2026-10-29

**Authors:** Yulu Zheng, Zheng Guo, Lei Zhao, Xingguo Peng, Yahong Zhu, Guangle Shan, Tingting Li, Xingang Li

**Affiliations:** ^1^ Centre for Precision Health Edith Cowan University Joondalup Australia; ^2^ Department of Otorhinolaryngology Heze Municipal Hospital Heze China; ^3^ Qinghai University Affiliated Hospital Xining China; ^4^ School of Science Edith Cowan University Joondalup Australia; ^5^ Department of Bioinformatics Thrive Bioresearch Beijing China; ^6^ Medical Research Center Jinan Maternity and Child Care Hospital Affiliated to Shandong First Medical University Jinan China

**Keywords:** cross‐sectional, hearing loss, later life, midlife, systemic inflammatory indices

## Abstract

**Objective:**

Systemic inflammatory indices, linked to immunosenescence‐related diseases, have not been thoroughly studied in hearing loss. This study aimed to determine whether systemic inflammatory indices are associated with hearing loss in midlife and later life.

**Methods:**

This serial cross‐sectional, community‐based investigation was conducted within the National Health and Nutrition Examination Survey 1999–2020 in United States. Hearing loss was defined as a pure‐tone average of ≥ 20 decibel hearing level at 0.5, 1, 2, and 4 kHz in the better ear. Six systemic inflammatory indices included systemic inflammation response index (SIRI), systemic immune‐inflammation index (SII), pan‐immune‐inflammation value (PIV), neutrophil lymphocyte ratio (NLR), platelet lymphocyte ratio (PLR), and lymphocyte monocyte ratio (LMR). Prevalence ratios (PRs) with 95% confidence intervals (CIs) derived from modified Poisson regression models (with adjustment for potential confounding factors) represented the associations between each systemic inflammatory index and hearing loss.

**Results:**

A total of 7920 adults aged 40–85 years were included. The mean (standard deviation) age was 58.6 (11.8) years, 4310 (54.4%) were women, and 2262 (28.6%) had hearing loss. After adjustment, hearing loss was associated with SIRI (PR 1.14, 95% CI, 1.08–1.20), SII (PR 1.07, 95% CI, 1.02–1.13), PIV (PR 1.11, 95% CI, 1.06–1.16), NLR (PR 1.10 95% CI, 1.04–1.17), and LMR (PR 0.87, 95% CI, 0.81–0.94).

**Conclusion:**

This study provides insights into the associations between systemic inflammatory indices and hearing loss, offering potential implications for risk stratification. While these indices are potential indicators for chronic inflammation in adults with hearing loss, further external validations are needed.

## 1 Introduction

Increased life expectancy has brought about a surge in age‐related adverse outcomes, and ageing intricately linked to diminished cognitive and sensory functions [1, 2]. Among the various sensory impairments affecting the elderly, hearing loss stands out as one of the most prevalent type [3]. A staggering 30% of middle‐aged adults and approximately 75% of the elderly grapple with varying degrees of hearing impairment, ranging from slight to profound [4, 5]. To enhance the overall quality of life and facilitate timely interventions for hearing loss, gaining a comprehensive understanding of the precise nature of hearing loss becomes imperative.

Peripheral hearing loss is typically categorized as either conductive or sensorineural [1]. Of these, sensorineural hearing loss emerges as the predominant type, constituting approximately 90% of all cases. Sensorineural hearing loss was acknowledged as a multifactorial condition where genetic risk factors, in conjunction with adverse environmental influences such as noise exposure, contribute to its development [6]. However, mechanistic insights of the sensorineural hearing loss remain largely unexplored. Recent evidence has increasingly supported the role of inflammation as a potential mechanism related to pathogenesis of hearing loss [7, 8, 9, 10].

Prolonged systemic chronic inflammation has been implicated in heightened risks of age‐related non‐communicable diseases (NCDs) or disorders, such as cardiovascular diseases (CVD) [11], dyslipidemia [12], neurodegenerative diseases [13], obesity [14], and type 2 diabetes [15]. These NCDs share a similar age‐dependent nature and have been linked with hearing loss [16, 17, 18, 19]. Systemic chronic inflammation could feasibly increase the risk of hearing loss through auditory neurological illness [9], or cochlear vascular damage [20]. Prior studies have explored associations between common inflammatory markers (such as interleukin‐6, C‐reactive protein, and tumor necrosis factor) and hearing loss, yielding inconsistent findings [9, 21, 22]. This inconsistency may stem from the fact that these markers are either acute phase reactants (APRs) or major mediators for the secretions of APRs. Unlike acute inflammation, low‐grade systemic chronic inflammation is non‐infective and involves the activation of immune components which are typically dissimilar from those engaged in the acute immune response [23, 24].

Current understanding of systemic inflammatory indices is increasingly comprehensive, for example, system inflammation response index (SIRI), systemic immune inflammation index (SII), pan‑immune‑inflammation value (PIV), neutrophil lymphocyte ratio (NLR), platelet lymphocyte ratio (PLR), and lymphocyte monocyte ratio (LMR). Those six well‐established indices integrating platelets and three independent white blood cell (WBC) subsets (neutrophils, monocytes, and lymphocytes). They reflect the intricate interplay of thrombocytosis, inflammation, and immunity [25]. Existing evidence has indicated the associations between these inflammatory indices and NCDs such as cognitive decline [25], CVD [26], ischemic stroke [27], hyperlipidemia [28], and gastric cancer [29]. However, to the best of our knowledge, comprehensive literature research revealed a dearth of studies exploring the associations between systemic inflammatory indices and hearing loss among adults.

Therefore, this study aims to fill this research gap by investigating whether well‐established systemic inflammatory indices are associated with hearing loss in mid‐ and later‐life.

## 2 Methods

### 2.1 Study Design and Participants

This serial cross‐sectional investigation was conducted within the framework of the National Health and Nutrition Examination Survey (NHANES). Comprehensive details regarding NHANES rationale, design, and recruitment can be found elsewhere [30]. In brief, NHANES is an ongoing annual survey initiated in 1999, designed to collect data through a complex, multistage, probability sample that accurately reflects the civilian, noninstitutionalized U.S. population. The data are released in 2‐year intervals, encompassing demographic, anthropometric, objective laboratory‐based, and subjective questionnaire‐based data.

For the current study, datasets spanning from 1999 to 2020 were utilized, merging data from the 1999 to 2000, 2001 to 2002, 2003 to 2004, 2005 to 2006, 2007 to 2008, 2009 to 2010, 2011 to 2012, 2015 to 2016, and 2017 to 2020 NHANES waves (audiometric data were not gathered in 2013 to 2014). Our final analytic cohort included 7920 adults with available data on audiometric test and modifiable risk factors (Figure 1).

**Figure 1:**
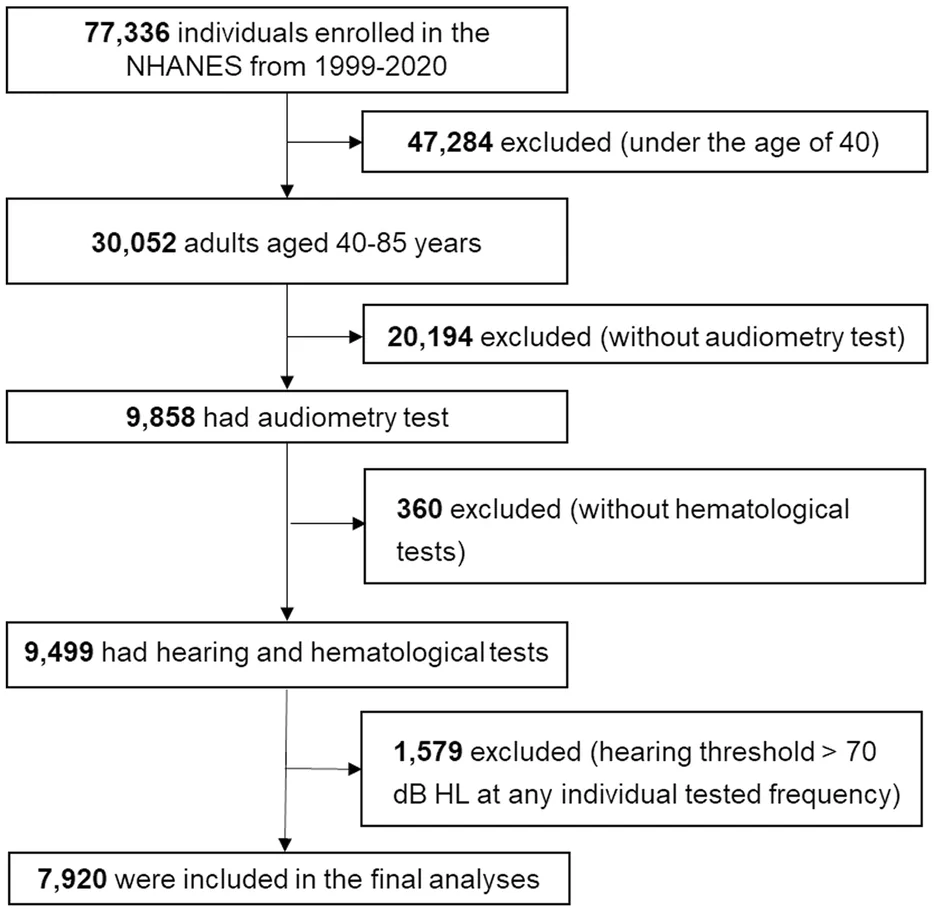
Participants flowchart. dB HL, decibel hearing level; NHANES, National Health and Nutrition Examination Survey.

All procedures received approval from the National Center for Health Statistics (NCHS) Ethics Review Board, and informed consent forms were obtained from all participants in this study (https://www.cdc.gov/nchs/nhanes/irba98.htm).

### 2.2 Hearing Assessment

Audiometry adhered to established NHANES protocols and was conducted in sound‐attenuating booths by a trained examiner. Hearing threshold testing encompassed both ears at frequencies 0.5, 1, 2, 3, 4, 6, and 8 kHz, utilizing a modified Hughson Westlake procedure. A four‐frequency (0.5, 1, 2, 4 kHz) pure‐tone average (PTA) was calculated for the better‐hearing ear, with higher scores denoting worse hearing. Additionally, high‐frequency (3, 4, 6, 8 kHz) and low‐frequency (0.5, 1, 2 kHz) PTAs were determined for the better‐hearing ear [31]. The primary outcome, hearing loss, was defined as a four‐frequency PTA ≥ 20 decibel hearing level (dB HL) in better ear [32].

### 2.3 Definitions of Six Systemic Inflammatory Indices

Six systemic inflammatory indices (SIRI [25, 27], SII [33], PIV [34], NLR [35], PLR [29], and LMR [36]) were calculated using the values of neutrophil count, monocyte count, lymphocyte count, and platelet count (presented as × 10^9^ cells/L). The calculations [25, 29, 33, 34, 35, 36] were listed as follows:

1. SIRI = (Neutrophil count × Monocyte count)/Lymphocyte count
2. SII = (Neutrophil count × Platelet count)/Lymphocyte count
3. PIV = (Neutrophil count × Monocyte count × Platelet count)/Lymphocyte count
4. NLR = Neutrophil count/Lymphocyte count
5. PLR = Platelet count/Lymphocyte count
6. LMR = Lymphocyte count/Monocyte count
7. xxx

Pairwise Spearman correlation coefficients were calculated among the six inflammatory indices to evaluate their correlation structure.

### 2.4 Statistical Analyses

Characteristics of the participants were summarized for the overall population. The correlation structure among the six inflammatory indices was evaluated using Spearman correlation coefficients. Given the violation of the rare disease assumption in this cross‐sectional design [37], modified Poisson regression models with sandwich estimations of variances were applied to ensure robust estimations for the associations of each of the log‐transformed inflammatory indices with hearing loss. Prevalence ratios (PRs) and their corresponding 95% confidence intervals (CIs) were assessed through three regression models: *Model 1* adjusted for age and sex; *Model 2* adjusted for age, sex, ethnicity, education attainment, and annual household income; *Model 3* further adjusted for hypertension, diabetes, body mass index (BMI), and non‐high‐density lipoprotein (non‐HDL) dyslipidemia based on *Model 2*. Selected potential confounding factors have been reported to be associated with hearing loss and/or chronic inflammation [5, 38, 39].

Variables with missing values and corresponding proportions before imputation included annual household income (11.6%), hypertension (1.5%), diabetes (0.2%), BMI (0.8%), and HDL‐C (2.1%). Multiple imputation (Multivariate Imputation via Chained Equations, “*MICE*” Package in R software version 4.1.0 [R Foundation for Statistical Computing, Vienna, Austria]) was employed to impute missing data. Assumed the data were missing at random, we utilized classification and regression trees (CART) as the conditional models for imputation [40]. Five imputations for 20 iterations were executed to ensure the convergence for all variables. Diagnostic statistics per estimate were computed by Rubin's rules [41].

Sensitivity analyses were conducted to verify robustness. First, we replicated the analyses for hearing loss at high‐ and low‐frequency PTA. Second, we reanalyzed the association between systemic inflammatory indices and more severe hearing loss, defined as the four‐frequency PTA > 40 dB HL [22]. Third, interaction terms between age group or sex and each inflammatory index were respectively included in the fully adjusted models to assess effect modification. Stratified analyses were subsequently performed according to age group and sex. Fourth, for future comparisons, we repeated analyses with the original value of systemic inflammatory indices. Fifth, self‐reported variables including smoking status, alcohol consumption were incorporated into the fully adjusted models. Sixth, survey‐weighted modified Poisson regression incorporating NHANES sampling weights, strata, and primary sampling units was conducted. Seventh, we repeated our primary association analysis (Model 3) by including all participants (*n* = 9499) regardless of hearing threshold severity. Last, four‐frequency PTA in the better‐hearing ear was additionally analyzed as a continuous outcome using multivariable linear regression with the same confounding variables adjustment strategy as the primary association analysis.

The analyses were performed using R software version 4.1.0 (R Foundation for Statistical Computing, Vienna, Austria). A significant level of *p* < 0.05 (2‐tailed) was considered throughout.

## 3 Results

### 3.1 Participants' Characteristics and Comparisons of Six Inflammatory Indices

Among the 7920 middle‐aged and elder participants aged 40–85 years, the mean age (standard deviation, SD) was 58.6 (11.8) years. Of these, 4310 were women (54.4%), and 3610 were men (45.6%). Hearing loss was reported in 2262 participants (28.6%) at four‐frequency PTA, in 4568 participants (57.7%) at high‐frequency PTA, and in 1,564 participants (19.7%) at low‐frequency PTA.

Detailed participants’ characteristics are provided in Table 1. Overall, individuals with hearing loss were older and reported lower levels of educational attainment and less annual household income compared to those with normal hearing. Statistically significant differences were observed in the prevalence of hypertension, diabetes, and non‐HDL dyslipidemia between hearing loss and normal hearing groups (*p* < 0.001). Violin plots in Figure 2 displayed the comparisons of six log‐transformed systemic inflammatory indices between adults with hearing loss and with normal hearing, showing statistical differences for SIRI, SII, PIV, NLR, and LMR, with no observed statistical difference for PLR. Moderate‐to‐high correlations were observed among several indices because they share overlapping hematological components (Figure S1).

**Table 1:** Characteristics of the study population.

Variables^a^	Normal hearing	Hearing loss^b^
Number pf participants, %	5658 (71.44)	2262 (28.56)
Age, mean (SD)	55.15 (10.43)	67.06 (10.62)^c^
Gender (Male, %)	2434 (43.0)	1176 (52.0)^c^
Ethnicity, %		
Mexican American	868 (15.3)	309 (13.7)^c^
Other Hispanic	543 (9.6)	207 (9.2)
Non‐Hispanic White	2123 (37.5)	1150 (50.8)
Non‐Hispanic Black	1463 (25.9)	418 (18.5)
Other Race	661 (11.7)	178 (7.9)
Education attainment, %		
Less than 12th grade	1296 (22.9)	767 (33.9)^c^
High school or GED education	1232 (21.8)	588 (26.0)
College or university education	3130 (55.3)	907 (40.1)
Annual family income, %		
< $35,000	2342 (41.4)	1196 (52.9)^c^
$35,000–$99,999	2519 (44.5)	897 (39.7)
> $100,000	797 (14.1)	169 (7.5)
Hypertension^d^, %	2891 (51.1)	1544 (68.3)^c^
Diabetes e, %	1054 (18.6)	694 (30.7)^c^
BMI f, mean (SD)	29.64 (6.87)	29.58 (6.50)
Non‐HDL dyslipidemia g, %	3612 (63.8)	1282 (56.7)^c^
Neutrophil count, median [IQR]	3.80 [3.00, 4.90]	4.05 [3.20, 5.10]^c^
Monocyte count, median [IQR]	0.50 [0.40, 0.60]	0.60 [0.40, 0.70]^c^
Lymphocyte count, median [IQR]	2.00 [1.60, 2.50]	1.90 [1.50, 2.40]^c^
Platelet count, median [IQR]	240.00 [204.00, 282.00]	229.00 [192.00, 270.00]^c^

Abbreviations: BMI, body mass index; GED, general educational development; IQR, interquartile range; non‐HDL: non‐high‐density lipoprotein; SD, standard deviation.

^a^ Comparisons of groups are based on *t* test for normally distributed continuous variables; *Mann‐Whitney U* test for non‐normally distributed continuous variables; *χ* ^*2*^ test for qualitative variables.

^b^ Defined as normal (PTA < 20 dB HL), and as slight to profound hearing loss (PTA ≥ 20 dB HL) for the better‐hearing ear at four frequency PTA (mean hearing threshold across 0.5, 1, 2 and 4 kHz.

^c^ *p* < 0.05.

^d^ Hypertension was defined as a blood pressure > 140/90 mmHg, a self‐reported history of hypertension, or treatment with antihypertensive medications.

^e^ Diabetes was defined as fasting glucose ≥ 7 mmol/L, or self‐reported history of diabetes, or treatment for diabetes.

^f^ BMI = weight (kg)/height (m)^2^_._

^g^ non‐HDL (total cholesterol minus HDL) dyslipidemia was defined as the value > 3.37 mmol/L.

**Figure 2:**
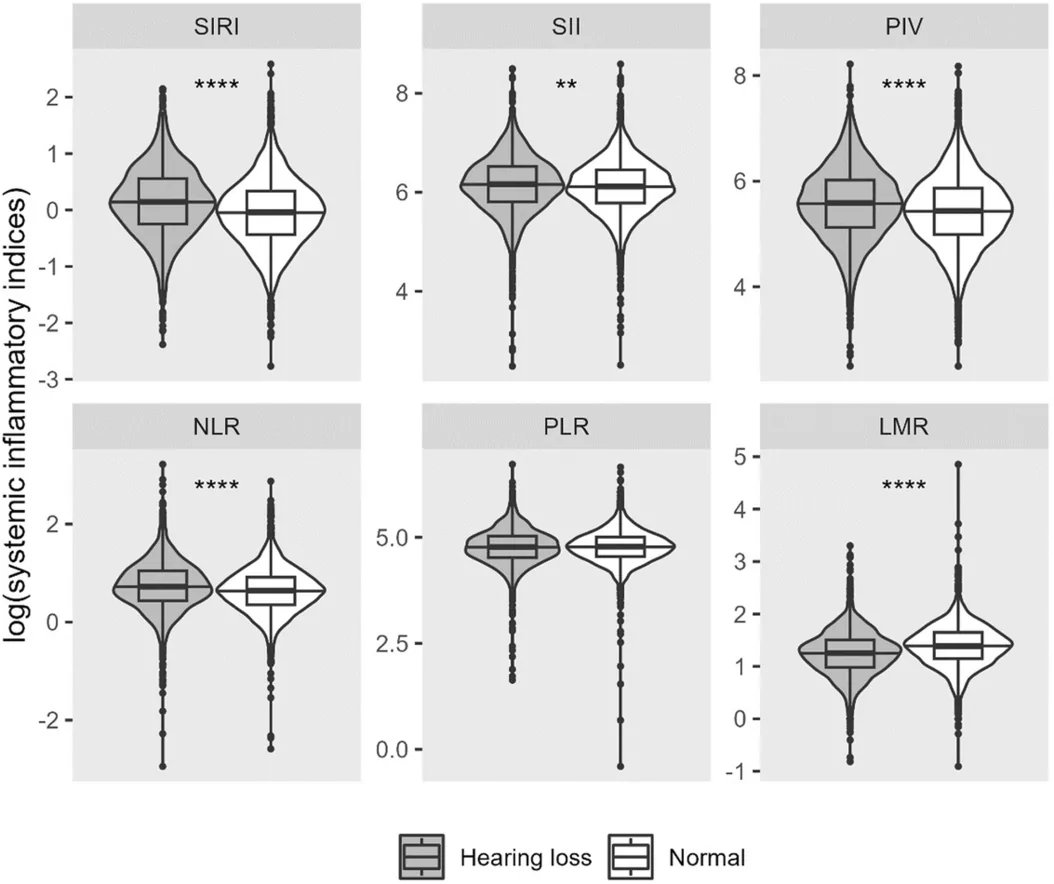
Comparisons of six log‐transformed systemic inflammatory indices between individuals with hearing loss and with normal hearing. **p < 0.01 and ****p < 0.0001. LMR, lymphocyte monocyte ratio; NLR, neutrophil lymphocyte ratio; PIV, pan‐immune‐inflammation value; PLR, platelet lymphocyte ratio; SII, systemic immune‐inflammation index; SIRI, systemic inflammation response index.

### 3.2 Associations of Six Systemic Inflammatory Indices and Hearing Loss

After adjusting for age, sex, ethnicity, education attainment, annual household income, hypertension, diabetes, BMI, and non‐HDL dyslipidemia, per‐unit increase in log‐transformed SIRI was associated with 14% increase in the prevalence of hearing loss (PR 1.14, 95% CI, 1.08, 1.20) compared with adults with normal hearing. Similar patterns of the associations were observed for the other three indices (SII, PIV, and NLR), while LMR showed a negative association with hearing loss. Per‐unit increase in log‐transformed LMR indicated a 13% decrease in the prevalence of hearing loss, with a PR of 0.87 (95% CI, 0.81, 0.94). No statistical association was found between PLR and hearing loss (Figure 3). All statistically significant associations remained significant after false discovery rate correction using the Benjamini‐Hochberg procedure.

**Figure 3:**
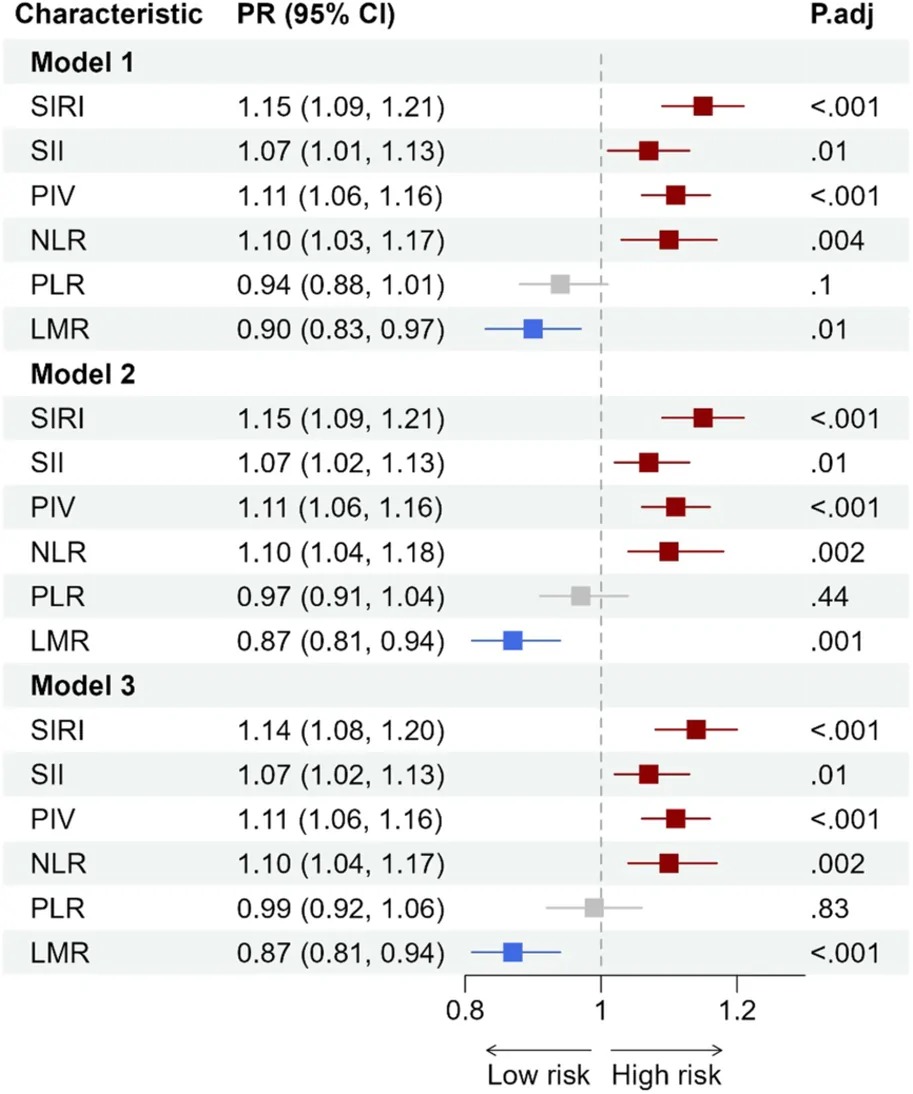
Associations of six systemic inflammatory indices and hearing loss, using modified Poisson regression. *Model 1* adjusted for age and sex; *Model 2* adjusted for age, sex, ethnicity, education attainment, and annual household income; *Model 3* further adjusted for hypertension, diabetes, body mass index, and non‐high‐density lipoprotein dyslipidemia based on *Model 2*. CI, confidence interval; LMR, lymphocyte monocyte ratio; NLR, neutrophil lymphocyte ratio; PIV, pan‐immune‐inflammation value; PLR, platelet lymphocyte ratio; PR, prevalence ratio; SII, systemic immune‐inflammation index; SIRI, systemic inflammation response index.

### 3.3 Sensitivity Analyses

Findings of sensitivity analyses were provided in the Supplementary Material. Similar cross‐sectional associations between three systemic inflammatory indices (SIRI, PIV, and NLR) and hearing loss were observed at high‐ and low‐ frequency PTA (Table S1). When assessing the associations for more severe hearing loss (four‐frequency PTA > 40 dB HL), only SIRI and LMR were associated with more severe hearing loss (Table S2). Formal interaction testing revealed significant age‐dependent modification of inflammatory index associations. Among the six indices examined, three showed statistically significant age group (40–64 vs. ≥ 65 years) × inflammatory index interactions: SIRI (interaction *β* = −0.23, *p* < 0.001), PIV (interaction *β* = −0.17, *p* < 0.001), and LMR (interaction *β* = 0.32, *p* < 0.001). These indicate attenuated associations in older adults, generally aligning with the subgroup findings (Table S3). Sensitivity analyses showed no significant sex interactions (interaction p‐values 0.09–0.71, Table S4), though associations were generally stronger among men than women. When using the original values of systemic inflammatory indices, associations were similar with the association patterns using the commonly applied log‐transformed systemic inflammatory indices (Table S5). Additional adjustment for smoking status, alcohol consumption, and noise exposure yielded materially unchanged results (all originally significant indices remained significant with consistent effect sizes; Table S6). Survey‐weighted analyses incorporating NHANES complex survey design produced results largely consistent with unweighted analyses, with primary associations maintaining consistent effect sizes (PRs) and directions (Table S7). When including all 9,499 individuals regardless of hearing threshold severity, association patterns remained consistent with the primary findings (Table S8). The direction of the associations between each systemic inflammatory index and continuous four‐frequency PTA and were consistent with that observed in the primary analyses based on the binary hearing‐loss outcome (Table S9).

## 4 Discussion

In the present study, we report associations between systemic inflammatory indices (SIRI, SII, PIV, NLR, and LMR) and the risk of hearing loss in a large, population‐based study spanning serial cross‐sectional NHANES data from 1999 to 2020. This translated into a unit increase in log‐transformed SIRI (or SII, PIV, NLR) was associated with a ~7% to ~14% increase in the prevalence of hearing loss, while one unit increase in log‐transformed LMR was associated with a ~13% decrease in the prevalence of hearing loss for mid‐life and older adults. However, it bore no associations of PLR with hearing loss in this investigation.

Formal interaction analyses demonstrated significant age‐related heterogeneity for SIRI, PIV, and LMR, with stronger associations observed among middle‐aged adults than older adults. This attenuation in older individuals may reflect the increasingly multifactorial nature of hearing loss, in which cumulative cochlear degeneration, vascular dysfunction, and lifetime environmental exposures become increasingly important determinants of hearing loss. In addition, age‐related chronic low‐grade inflammation (“inflammaging”) may reduce the discriminatory ability of inflammatory markers in older populations [11]. Although several associations appeared stronger in men, sex interactions were not statistically significant, suggesting that these findings should be interpreted cautiously. Nevertheless, sex differences in immune responses and hormonal regulation may partly contribute to the observed patterns [42].

Chronic inflammation, often referred to as inflammaging, plays a pivotal role in age‐related diseases [25, 43]. While the mechanisms of hearing loss are not fully understood, plausible explanations potentially also involved inflammation [44]. Research suggests that as individuals age, specific lymphocyte populations, namely interleukin 1 receptor type II‐positive T cells and naturally occurring regulatory T cells in CD4^+^ T cells, contribute to suppressing host immune function and promoting organ degeneration, including cochlear [45]. Notably, chronic inflammation, represented by the WBC count, has been linked to age‐related hearing loss among community‐dwelling adults aged over 75 years [8]. This underscores the potential role of chronic and low‐grade inflammation and hearing loss [9].

WBCs, implicated in tissue damage following ischemic changes or stroke, may contribute to tissue damage in the inner ear, leading to hearing loss [27, 46, 47]. The six systemic inflammatory indices used in this study (SIRI, SII, PIV, NLR, PLR, and LMR) were calculated based on platelets and three WBC subtypes (neutrophils, monocytes, and lymphocytes). Platelets are known contributors to thrombosis, coagulation, inflammation, and atherosclerosis [48]. Neutrophils can exacerbate vessel wall inflammation [49], correlate with the risk of plaque rupture, and increase the risk of thrombosis in the microcirculation [50]. Monocytes play a crucial role in initiating and promoting the progression of chronic inflammatory disease through the release of pro‐inflammatory cytokines [51]. Furthermore, an animal‐based study revealed that the aggravated hearing loss after noise exposure is associated with monocyte infiltration in the cochlea [52]. Conversely, lymphocytes impede the progression of chronic inflammatory disease, such as atherosclerosis [26]. In a senescence‐associated animal model, lymphocytes from young mice were found to prevent the progression of age‐related hearing loss [53].

Our study is advantaged in the appropriateness of the modified Poisson regression models, providing unbiased estimates for prevalence ratios in the case of the binary outcome. Additionally, the investigation included large sample size, formal interaction testing, and multiple sensitivity analyses demonstrating robustness. Moreover, to our knowledge, few studies have comprehensively evaluated multiple composite inflammatory indices in relation to hearing loss, and no previous study has simultaneously examined SIRI, SII, PIV, NLR, PLR, and LMR in a nationally representative population of middle‐aged and older adults. While the mechanisms of hearing loss are not thoroughly understood, our findings contribute to a theoretical foundation and suggest directions for future research to delve into the association of chronic inflammation with hearing loss.

Despite these strengths, our study has limitations. The cross‐sectional nature impedes the establishment of causality or the directionality of the association between systemic inflammatory indices and hearing loss. Furthermore, potential underestimation may exist as participants with disadvantaged socioeconomic backgrounds were less represented, a group that may exhibit a higher prevalence of both inflammation and hearing loss. Additionally, while we adjusted for major well‐established confounders, residual confounding cannot be excluded. This limitation primarily stems from the lack of standardized or uniform data across all NHANES cycles regarding critical variables, including ototoxic medication use, cardiovascular history, acute infections, hematological disorders, and malignancies, all of which could transiently or fundamentally alter the studied indices. Future investigations should adopt longitudinal designs with the consideration of gene‐environmental interactions, and causal effect studies, to better understand the pathogenesis of hearing loss. This could determine the causality and directionality of the association between systemic chronic inflammation and hearing loss.

While our study focused on hearing loss in the general population, systemic inflammatory indices may have different clinical relevance across distinct hearing loss phenotypes. For instance, recent evidence suggests that inflammatory markers show promise as prognostic indicators in acute presentations such as sudden sensorineural hearing loss [54, 55]. Additionally, bilateral and immune‐mediated hearing loss may exhibit different inflammatory profiles compared to unilateral or age‐related presentations [54]. Future prospective studies should evaluate whether systemic inflammatory indices, particularly when integrated with audiometric and clinical variables, could serve as components of classification models not only for hearing loss risk stratification but also for prognosis and recovery outcomes in various hearing loss phenotypes.

## 5 Conclusions

This study provides insights into the associations between systemic inflammatory indices and hearing loss, offering potential implications for risk stratification. The study reveals that individuals with hearing loss exhibit a decreased level of systemic inflammatory index LMR and elevated levels of systemic inflammatory indices SIRI, SII, PIV, and NLR compared to those with normal hearing. While these indices represent potential indicators for chronic inflammation in adults with hearing loss, their causal relationships and clinical utility warrant further investigation through longitudinal studies and external validations.

## Author Contributions

**Yulu Zheng:** formal analysis, methodology, software, writing – original draft, writing – review and editing. **Zheng Guo:** conceptualization, data curation, writing – original draft. **Lei Zhao, Xingguo Peng, Yahong Zhu,** and **Guangle Shan:** writing – review and editing. **Tingting Li:** conceptualization, supervision, writing – review and editing. **Xingang Li:** conceptualization, supervision, funding acquisition, writing – review and editing.

## Ethics Statement

All procedures received approval from the National Center for Health Statistics (NCHS) Ethics Review Board, and informed consent forms were obtained from all participants in this study (https://www.cdc.gov/nchs/nhanes/irba98.htm).

## Conflicts of Interest

The authors declare no conflicts of interest.

## Supporting information


**Figure S1:** Spearman correlation matrix of the six inflammatory indices.
**Table S1:** Associations between systemic inflammatory indices and hearing loss in high‐ and low‐frequency.
**Table S2:** Associations between systemic inflammatory indices and more severe hearing loss.
**Table S3:** Associations between systemic inflammatory indices and hearing loss, stratified by age.
**Table S4:** Associations between systemic inflammatory indices and hearing loss, stratified by sex.
**Table S5:** Associations between original systemic inflammatory indices and hearing loss.
**Table S6:** Associations between systemic inflammatory indices and hearing loss, with additional adjustment for smoking status, alcohol drinking, and noise exposure.
**Table S7:** Survey‐weighted associations between systemic inflammatory indices and hearing loss, with additional adjustment for smoking status, alcohol drinking, and noise exposure.
**Table S8:** Associations between systemic inflammatory indices and hearing loss in 9,499 adults.Table S9: Associations between systemic inflammatory indices and four frequency PTA.

## Data Availability

The data that support the findings of this study are openly available in Questionnaires, Datasets, and Related Documentation at https://www.cdc.gov/nchs/nhanes/.
