# Supplementary material for: Systemic Inflammatory Indices and Hearing Loss in U.S. Adults Aged 40–85 Years: A Serial Cross‐Sectional Study 1999–2020
Source: Immun Inflamm Dis. 2026 Oct 29;14(10):e70576. doi: 10.1002/iid3.70576 (PMC13720236; doi:10.1002/iid3.70576)
Supplement: Supplementary file 1 [file IID3-14-e70576-s001.docx]

**Supplementary Material**

**Systemic Inflammatory Indices and Hearing Loss in U.S. Adults Aged 40-85 Years: A Serial Cross-sectional Study 1999-2020**

Yulu Zheng^1,#^, Zheng Guo^1,#^, Lei Zhao^2^, Xingguo Peng^3^, Yahong Zhu^4^, Guangle Shan^5^, Tingting Li ^6,^*, and Xingang Li ^1,^*

^#^**Yulu Zheng** and **Zheng Guo** contributed equally to this work.

^1^ Centre for Precision Health, Edith Cowan University, Joondalup, Australia.

^2^ Department of Otorhinolaryngology, Heze Municipal Hospital, Heze, China.

^3^ Qinghai University Affiliated Hospital, Xining, China.

^4^ School of Science, Edith Cowan University, Joondalup, Australia.

^5^ Department of Bioinformatics, Thrive Bioresearch, China.

^6^ Medical Research Center, Jinan Maternity and Child Care Hospital Affiliated to Shandong First Medical University, Jinan, China.

***Corresponding Authors**:

**Tingting Li** (litingtingtt@126.com)

**Xingang Li** (xingang.li@ecu.edu.au)

**Figure S1 Spearman correlation matrix of the six inflammatory indices**

Spearman correlation coefficients were calculated among SIRI, SII, PIV, NLR, PLR, and LMR. Positive correlations are shown in yellow and negative correlations in purple.

*Abbreviations:* *SIRI*, systemic inflammation response index; *SII*, systemic immune-inflammation index; *PIV*, pan-immune-inflammation value; *NLR*, neutrophil lymphocyte ratio; *PLR*, platelet lymphocyte ratio; *LMR*, lymphocyte monocyte ratio.

**Table S1 Associations between systemic inflammatory indices and hearing loss in high- and low-frequency**

| **Hearing loss ^a^** | **PR** | **Lower CI** | **Upper CI** | ***P*** |
| --- | --- | --- | --- | --- |
| ***High-frequency PTA* ^b^** |  |  |  |  |
| SIRI | 1.07 | 1.04 | 1.09 | <0.001 |
| SII | 1.04 | 1.01 | 1.07 | 0.007 |
| PIV | 1.05 | 1.03 | 1.07 | <0.001 |
| NLR | 1.05 | 1.02 | 1.09 | 0.001 |
| PLR | 0.99 | 0.96 | 1.03 | 0.64 |
| LMR | 0.94 | 0.91 | 0.98 | 0.003 |
| ***Low-frequency PTA* ^b^** |  |  |  |  |
| SIRI | 1.12 | 1.04 | 1.20 | 0.002 |
| SII | 1.07 | 0.99 | 1.15 | 0.06 |
| PIV | 1.08 | 1.02 | 1.14 | 0.01 |
| NLR | 1.11 | 1.03 | 1.21 | 0.01 |
| PLR | 0.99 | 0.91 | 1.09 | 0.89 |
| LMR | 0.90 | 0.82 | 1.01 | 0.06 |

^a^ Defined as the threshold ≥ 20 *dB HL* in the better-hearing ear

^b^ **High-frequency PTA**: 3, 4, 6, and 8 kHz; **Low-frequency PTA**: 0.5, 1, and 2 kHz.

The systemic inflammatory indices were log-transformed, and the regression models were adjusted for age, sex, ethnicity, education, income, hypertension, diabetes, body mass index, and non-HDL dyslipidemia.

*Abbreviations:* *PR*, prevalence ratio; *CI*, confidence interval; *PTA*, pure-tone average; *SIRI*, systemic inflammation response index; *SII*, systemic immune-inflammation index; *PIV*, pan-immune-inflammation value; *NLR*, neutrophil lymphocyte ratio; *PLR*, platelet lymphocyte ratio; *LMR*, lymphocyte monocyte ratio.

**Table S2** **Associations between systemic inflammatory indices and more severe hearing loss**

| **More severe hearing loss ^a^** | **PR** | **Lower CI** | **Upper CI** | ***P*** |
| --- | --- | --- | --- | --- |
| SIRI | 1.25 | 1.04 | 1.51 | 0.02 |
| SII | 1.09 | 0.89 | 1.32 | 0.41 |
| PIV | 1.12 | 0.94 | 1.33 | 0.19 |
| NLR | 1.24 | 0.99 | 1.55 | 0.06 |
| PLR | 1.07 | 0.84 | 1.36 | 0.60 |
| LMR | 0.68 | 0.51 | 0.89 | 0.01 |

^a^ Defined as the threshold > 40 *dB HL* in the better-hearing ear at four frequency PTA (mean hearing threshold across 0.5, 1, 2 and 4 kHz).

The systemic inflammatory indices were log-transformed, and the regression models were adjusted for age, sex, ethnicity, education, income, hypertension, diabetes, body mass index, non-HDL dyslipidemia.

*Abbreviations: PR*, prevalence ratio; *CI*, confidence interval; *PTA*, pure-tone average; *SIRI*, systemic inflammation response index; *SII*, systemic immune-inflammation index; *PIV*, pan-immune-inflammation value; *NLR*, neutrophil lymphocyte ratio; *PLR*, platelet lymphocyte ratio; *LMR*, lymphocyte monocyte ratio.

**Table S3 Associations between systemic inflammatory indices and hearing loss, stratified by age**

| **Hearing loss ^a^** | **Middle-aged adults ^b^** | | **Older adults ^b^** | | **P for interaction (Ref: Middle-aged) ^c^** |
| --- | --- | --- | --- | --- | --- |
|  | **PR (95% CI)** | **P** | **PR (95% CI)** | **P** | **P_inflammatory_index_Older** |
| SIRI | 1.28 (1.16, 1.41) | <0.001 | 1.08 (1.02, 1.14) | 0.01 | <0.001 |
| SII | 1.14 (1.02, 1.27) | 0.03 | 1.05 (0.99, 1.11) | 0.12 | 0.25 |
| PIV | 1.22 (1.12, 1.33) | <0.001 | 1.05 (1.00, 1.1) | 0.06 | 0.001 |
| NLR | 1.16 (1.02, 1.32) | 0.02 | 1.08 (1.01, 1.15) | 0.02 | 0.12 |
| PLR | 0.98 (0.83, 1.14) | 0.76 | 0.99 (0.92, 1.07) | 0.88 | 0.35 |
| LMR | 0.74 (0.64, 0.86) | <0.001 | 0.94 (0.87, 1.02) | 0.16 | <0.001 |

^a^ Defined as the threshold ≥ 20 *dB HL* in the better-hearing ear at four frequency PTA (mean hearing threshold across 0.5, 1, 2 and 4 kHz).

^b^ Middle-aged adults: aged 40-64 years old; older adults: aged ≥ 65 years old.

^c^ P for interaction was calculated by including systemic inflammatory index × age group interaction term based on Model 3 among all adults.

The systemic inflammatory indices were log-transformed, and the regression models were adjusted for age, sex, ethnicity, education, income, hypertension, diabetes, body mass index, and non-HDL dyslipidemia.

*Abbreviations:* *PR*, prevalence ratio; *CI*, confidence interval; *PTA*, pure-tone average; *SIRI*, systemic inflammation response index; *SII*, systemic immune-inflammation index; *PIV*, pan-immune-inflammation value; *NLR*, neutrophil lymphocyte ratio; *PLR*, platelet lymphocyte ratio; *LMR*, lymphocyte monocyte ratio.

**Table S4** **Associations between systemic inflammatory indices and hearing loss, stratified by sex**

| **Hearing loss ^a^** | **Female** | | **Male** | | **P for interaction (Ref: Male) ^b^** |
| --- | --- | --- | --- | --- | --- |
|  | **PR (95% CI)** | **P** | **PR (95% CI)** | **P** | **P_inflammatory_index_Female** |
| SIRI | 1.06 (0.98, 1.15) | 0.12 | 1.2 (1.12, 1.28) | <0.001 | 0.09 |
| SII | 1.03 (0.95, 1.11) | 0.48 | 1.11 (1.03, 1.19) | 0.01 | 0.24 |
| PIV | 1.05 (0.98, 1.12) | 0.18 | 1.15 (1.08, 1.22) | <0.001 | 0.09 |
| NLR | 1.04 (0.95, 1.14) | 0.41 | 1.15 (1.05, 1.25) | <0.001 | 0.26 |
| PLR | 0.96 (0.86, 1.07) | 0.44 | 1.01 (0.92, 1.11) | 0.79 | 0.71 |
| LMR | 0.97 (0.86, 1.09) | 0.57 | 0.81 (0.73, 0.89) | <0.001 | 0.22 |

^a^ Defined as the threshold ≥ 20 *dB HL* in the better-hearing ear at four frequency PTA (mean hearing threshold across 0.5, 1, 2 and 4 kHz).

^b^ P for interaction was calculated by including systemic inflammatory index × sex interaction term based on Model 3 among all adults.

The systemic inflammatory indices were log-transformed, and the regression models were adjusted for age, sex, ethnicity, education, income, hypertension, diabetes, body mass index, and non-HDL dyslipidemia.

*Abbreviations:* *PR*, prevalence ratio; *CI*, confidence interval; *PTA*, pure-tone average; *SIRI*, systemic inflammation response index; *SII*, systemic immune-inflammation index; *PIV*, pan-immune-inflammation value; *NLR*, neutrophil lymphocyte ratio; *PLR*, platelet lymphocyte ratio; *LMR*, lymphocyte monocyte ratio.

**Table S5** **Associations between original systemic inflammatory indices and hearing loss**

| **Hearing loss ^a^** | **PR** | **Lower CI** | **Upper CI** | ***P*** |
| --- | --- | --- | --- | --- |
| SIRI | 1.07 | 1.04 | 1.10 | <0.001 |
| SII | 1.01 | 1.01 | 1.01 | <0.001 |
| PIV | 1.01 | 1.01 | 1.01 | <0.001 |
| NLR | 1.04 | 1.02 | 1.06 | <0.001 |
| PLR | 0.99 | 0.99 | 1.01 | 0.67 |
| LMR | 0.98 | 0.96 | 0.99 | 0.01 |

^a^ Defined as the threshold ≥ 20 *dB HL* in the better-hearing ear at four frequency PTA (mean hearing threshold across 0.5, 1, 2 and 4 kHz).

The regression models were adjusted for age, sex, ethnicity, education, income, hypertension, diabetes, body mass index, and non-HDL dyslipidemia.

*Abbreviations:* *PR*, prevalence ratio; *CI*, confidence interval; *PTA*, pure-tone average; *SIRI*, systemic inflammation response index; *SII*, systemic immune-inflammation index; *PIV*, pan-immune-inflammation value; *NLR*, neutrophil lymphocyte ratio; *PLR*, platelet lymphocyte ratio; *LMR*, lymphocyte monocyte ratio.

**Table S6 Associations between systemic inflammatory indices and hearing loss, with additional adjustment for smoking status, alcohol drinking, and noise exposure**

| **Hearing loss ^a^** | **PR** | **Lower CI** | **Upper CI** | ***P*** |
| --- | --- | --- | --- | --- |
| SIRI | 1.14 | 1.08 | 1.2 | <0.001 |
| SII | 1.08 | 1.02 | 1.13 | <0.001 |
| PIV | 1.10 | 1.05 | 1.15 | <0.001 |
| NLR | 1.11 | 1.04 | 1.18 | 0.002 |
| PLR | 1.01 | 0.94 | 1.08 | 0.88 |
| LMR | 0.86 | 0.80 | 0.93 | <0.001 |

^a^ Defined as the threshold ≥ 20 *dB HL* in the better-hearing ear at four frequency PTA (mean hearing threshold across 0.5, 1, 2 and 4 kHz).

The regression models were adjusted for age, sex, ethnicity, education, income, hypertension, diabetes, body mass index, non-HDL dyslipidemia, smoking status, alcohol drinking, and noise exposure.

*Abbreviations:* *PR*, prevalence ratio; *CI*, confidence interval; *PTA*, pure-tone average; *SIRI*, systemic inflammation response index; *SII*, systemic immune-inflammation index; *PIV*, pan-immune-inflammation value; *NLR*, neutrophil lymphocyte ratio; *PLR*, platelet lymphocyte ratio; *LMR*, lymphocyte monocyte ratio.

**Table S7 Survey-weighted associations between systemic inflammatory indices and hearing loss, with additional adjustment for smoking status, alcohol drinking, and noise exposure**

| **Hearing loss ^a^** | **Weighted PR** | **Lower CI** | **Upper CI** | ***P*** |
| --- | --- | --- | --- | --- |
| SIRI | 1.12 | 1.05 | 1.2 | 0.001 |
| SII | 1.10 | 1.01 | 1.19 | 0.03 |
| PIV | 1.11 | 1.05 | 1.18 | <0.001 |
| NLR | 1.09 | 0.99 | 1.21 | 0.09 |
| PLR | 1.03 | 0.93 | 1.15 | 0.56 |
| LMR | 0.91 | 0.82 | 1.02 | 0.10 |

^a^ Defined as the threshold ≥ 20 *dB HL* in the better-hearing ear at four frequency PTA (mean hearing threshold across 0.5, 1, 2 and 4 kHz).

The survey-weighted modified Poisson regression models were adjusted for age, sex, ethnicity, education, income, hypertension, diabetes, body mass index, non-HDL dyslipidemia, smoking status, alcohol drinking, and noise exposure.

*Abbreviations:* *PR*, prevalence ratio; *CI*, confidence interval; *PTA*, pure-tone average; *SIRI*, systemic inflammation response index; *SII*, systemic immune-inflammation index; *PIV*, pan-immune-inflammation value; *NLR*, neutrophil lymphocyte ratio; *PLR*, platelet lymphocyte ratio; *LMR*, lymphocyte monocyte ratio.

**Table S8 Associations between systemic inflammatory indices and hearing loss in 9,499 adults**

| **Hearing loss ^a^** | **PR** | **Lower CI** | **Upper CI** | ***P*** |
| --- | --- | --- | --- | --- |
| SIRI | 1.12 | 1.07 | 1.17 | <0.0001 |
| SII | 1.05 | 1.01 | 1.10 | 0.04 |
| PIV | 1.09 | 1.05 | 1.13 | <0.0001 |
| NLR | 1.07 | 1.02 | 1.13 | 0.01 |
| PLR | 0.97 | 0.92 | 1.03 | 0.37 |
| LMR | 0.88 | 0.83 | 0.94 | <0.001 |

^a^ Defined as the threshold ≥ 20 *dB HL* in the better-hearing ear at four frequency PTA (mean hearing threshold across 0.5, 1, 2 and 4 kHz).

Modified Poisson regression models were applied, adjusted for age, sex, ethnicity, education, income, hypertension, diabetes, body mass index, and non-HDL dyslipidemia.

*Abbreviations:* *PR*, prevalence ratio; *CI*, confidence interval; *PTA*, pure-tone average; *SIRI*, systemic inflammation response index; *SII*, systemic immune-inflammation index; *PIV*, pan-immune-inflammation value; *NLR*, neutrophil lymphocyte ratio; *PLR*, platelet lymphocyte ratio; *LMR*, lymphocyte monocyte ratio.

**Table S9 Associations between systemic inflammatory indices and four frequency PTA**

| **Four frequency PTA** | **β** | **Lower CI** | **Upper CI** | ***P*** |
| --- | --- | --- | --- | --- |
| SIRI | 1.33 | 1.00 | 1.67 | <0.0001 |
| SII | 0.86 | 0.49 | 1.23 | <0.0001 |
| PIV | 1.04 | 0.75 | 1.34 | <0.0001 |
| NLR | 1.12 | 0.69 | 1.55 | <0.0001 |
| PLR | -0.32 | -0.84 | 0.21 | 0.24 |
| LMR | -1.13 | -1.65 | -0.61 | <0.0001 |

Linear regression models were used to assess the associations between each index and four frequency PTA (mean hearing threshold across 0.5, 1, 2 and 4 kHz, better ear).

*Abbreviations:* *PR*, prevalence ratio; *CI*, confidence interval; *PTA*, pure-tone average; *SIRI*, systemic inflammation response index; *SII*, systemic immune-inflammation index; *PIV*, pan-immune-inflammation value; *NLR*, neutrophil lymphocyte ratio; *PLR*, platelet lymphocyte ratio; *LMR*, lymphocyte monocyte ratio.
